# 3-Acetyl-1,5-diphenyl-1*H*-pyrazole-4-carbonitrile

**DOI:** 10.1107/S1600536812010938

**Published:** 2012-03-17

**Authors:** Hatem A. Abdel-Aziz, Hazem A. Ghabbour, Suchada Chantrapromma, Hoong-Kun Fun

**Affiliations:** a Department of Pharmaceutical Chemistry, College of Pharmacy, King Saud University, PO Box 2457, Riyadh 11451, Saudi Arabia; b Crystal Materials Research Unit, Department of Chemistry, Faculty of Science, Prince of Songkla University, Hat-Yai, Songkhla 90112, Thailand; c X-ray Crystallography Unit, School of Physics, Universiti Sains Malaysia, 11800 USM, Penang, Malaysia

## Abstract

The title compound, C_18_H_13_N_3_O, has a butterfly-like structure, in which the pyrazole ring forms dihedral angles of 59.31 (8) and 57.24 (8)° with the two phenyl rings. The dihedral angle between the two phenyl rings is 64.03 (8)°. The pyrazole ring and the C—C=O plane of the acetyl group are twisted slightly, making a dihedral angle of 7.95 (18)°. In the crystal, mol­ecules are linked through weak C—H⋯N and C—H⋯O inter­actions into a helical chain along the *a*-axis direction.

## Related literature

For bond-length data, see: Allen *et al.* (1987 ▶). For background to and the bioactivity of pyrazole derivatives, see: Abdel-Aziz *et al.* (2009 ▶, 2010 ▶); Abdel-Wahab *et al.* (2009 ▶); Bharate *et al.* (2008 ▶); Dawood *et al.* (2003 ▶); Fu *et al.* (2010 ▶); Thumar & Patel (2011 ▶). For a related structure, see: Abdel-Aziz *et al.* (2011 ▶).
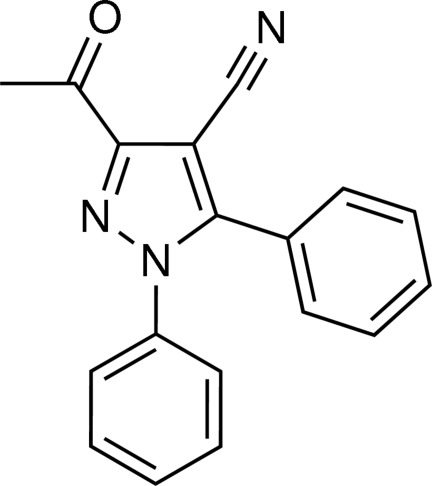


## Experimental

### 

#### Crystal data

C_18_H_13_N_3_O
*M* *_r_* = 287.31
Orthorhombic, 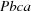

*a* = 6.8322 (2) Å
*b* = 16.8974 (5) Å
*c* = 25.7968 (6) Å
*V* = 2978.15 (14) Å^3^
*Z* = 8
Cu *K*α radiation
μ = 0.66 mm^−1^
*T* = 296 K
0.56 × 0.35 × 0.23 mm

#### Data collection

Bruker SMART APEXII CCD area-detector diffractometer
Absorption correction: multi-scan (*SADABS*; Bruker, 2009 ▶) *T*_min_ = 0.708, *T*_max_ = 0.863
10678 measured reflections
2782 independent reflections
2338 reflections with *I* > 2σ(*I*)
*R*_int_ = 0.026

#### Refinement

*R*[*F* ^2^ > 2σ(*F* ^2^)] = 0.039
*wR*(*F* ^2^) = 0.116
*S* = 1.08
2782 reflections
201 parameters
H-atom parameters constrained
Δρ_max_ = 0.18 e Å^−3^
Δρ_min_ = −0.17 e Å^−3^

### 

Data collection: *APEX2* (Bruker, 2009 ▶); cell refinement: *SAINT* (Bruker, 2009 ▶); data reduction: *SAINT*; program(s) used to solve structure: *SHELXTL* (Sheldrick, 2008 ▶); program(s) used to refine structure: *SHELXTL*; molecular graphics: *SHELXTL*; software used to prepare material for publication: *SHELXTL* and *PLATON* (Spek, 2009 ▶).

## Supplementary Material

Crystal structure: contains datablock(s) global, I. DOI: 10.1107/S1600536812010938/is5091sup1.cif

Structure factors: contains datablock(s) I. DOI: 10.1107/S1600536812010938/is5091Isup2.hkl

Supplementary material file. DOI: 10.1107/S1600536812010938/is5091Isup3.cml

Additional supplementary materials:  crystallographic information; 3D view; checkCIF report

## Figures and Tables

**Table 1:** Hydrogen-bond geometry (Å, °)

*D*—H⋯*A*	*D*—H	H⋯*A*	*D*⋯*A*	*D*—H⋯*A*
C6—H6*A*⋯N3^i^	0.93	2.53	3.432 (2)	165
C16—H16*A*⋯O1^ii^	0.93	2.59	3.3758 (19)	142

Symmetry codes: (i) 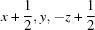; (ii) 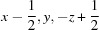.

**Fig. 1.**
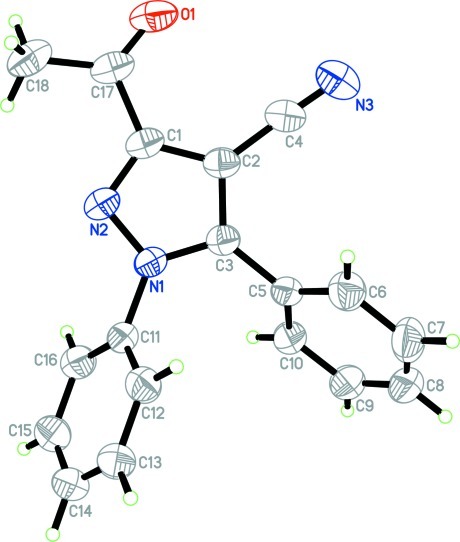
The molecular structure of the title compound, showing 40% probability displacement ellipsoids and the atom-numbering scheme.

**Fig. 2.**
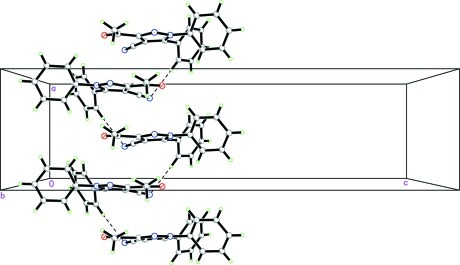
The crystal packing diagram of the title compound viewed along the b axis, showing the helical chain along the [1 0 0]. C—H···N hydrogen bonds are shown as dashed lines.

## supplementary crystallographic information

### Comment

Owing to the various biological properties of pyrazole derivatives such as anti-cancer (Fu *et al.*, 2010), anti-inflammatory (Bharate *et al.*, 2008) and antimicrobial activities (Thumar & Patel, 2011), we have during the course of our medicinal chemistry research reported the synthesis and bioactivity of pyrazole derivatives (Abdel-Aziz *et al.*, 2009, 2010; Abdel-Wahab *et al.*, 2009). The title compound (I) was synthesized and characterized in order to study the structure activity relationship of this class of compounds.

The molecule of (I), C_18_H_13_N_3_O, has a butterfly-like structure. The pyrazole ring forms the dihedral angles of 59.31 (8) and 57.24 (8)°, respectively, with the C5–C10 and C11–C16 phenyl rings, whereas the dihedral angle between these two rings is 64.03 (8)°. The cabonitrile substituent lies on the same plane with the pyrazole ring with an *r.m.s*. 0.0027 (1) Å for the seven non-H atoms (C1–C4/N1–N3), whereas the acetyl group is slightly deviated with the torsion angles N2–C1–C17–C18 = 8.3 (2)° and N2–C1–C17–O1 = -171.47 (13)°. The bond distances in (I) are within normal ranges (Allen *et al.*, 1987) and comparable to the related structure (Abdel-Aziz *et al.*, 2011). The crystal packing of (I) is stabilized by weak C—H···N and C—H···O interactions (Table 1). Figure 2 shows the molecular a helical chain along the [1 0 0] linked by these interactions.

### Experimental

The title compound was prepared according to the reported method (Dawood *et al.*, 2003). Single crystals of the title compound suitable for X-ray structure determination were recrystallized from ethanol by the slow evaporation of the solvent at room temperature after several days.

### Refinement

All H atoms were placed in calculated positions with d(C—H) = 0.93 for aromatic and 0.96 Å for CH_3_ atoms. The *U*_iso_(H) values were constrained to be 1.5*U*_eq_ of the carrier atom for methyl H atoms and 1.2*U*_eq_ for the remaining H atoms. A rotating group model was used for the methyl groups.

### Crystal data

**Table:**

C_18_H_13_N_3_O	*F*(000) = 1200
*M**_r_* = 287.31	*D*_x_ = 1.282 Mg m^−^^3^
Orthorhombic, *P**b**c**a*	Cu *K*α radiation, λ = 1.54178 Å
Hall symbol: -P 2ac 2ab	Cell parameters from 2782 reflections
*a* = 6.8322 (2) Å	θ = 3.4–69.9°
*b* = 16.8974 (5) Å	µ = 0.66 mm^−^^1^
*c* = 25.7968 (6) Å	*T* = 296 K
*V* = 2978.15 (14) Å^3^	Block, colorless
*Z* = 8	0.56 × 0.35 × 0.23 mm

### Data collection

**Table:**

Bruker SMART APEXII CCD area-detector diffractometer	2782 independent reflections
Radiation source: sealed tube	2338 reflections with *I* > 2σ(*I*)
Graphite monochromator	*R*_int_ = 0.026
φ and ω scans	θ_max_ = 69.9°, θ_min_ = 3.4°
Absorption correction: multi-scan (*SADABS*; Bruker, 2009)	*h* = −6→8
*T*_min_ = 0.708, *T*_max_ = 0.863	*k* = −20→20
10678 measured reflections	*l* = −31→23

### Refinement

**Table:**

Refinement on *F*^2^	Secondary atom site location: difference Fourier map
Least-squares matrix: full	Hydrogen site location: inferred from neighbouring sites
*R*[*F*^2^ > 2σ(*F*^2^)] = 0.039	H-atom parameters constrained
*wR*(*F*^2^) = 0.116	*w* = 1/[σ^2^(*F*_o_^2^) + (0.0586*P*)^2^ + 0.4109*P*] where *P* = (*F*_o_^2^ + 2*F*_c_^2^)/3
*S* = 1.08	(Δ/σ)_max_ = 0.001
2782 reflections	Δρ_max_ = 0.18 e Å^−^^3^
201 parameters	Δρ_min_ = −0.17 e Å^−^^3^
0 restraints	Extinction correction: *SHELXTL* (Sheldrick, 2008), Fc^*^=kFc[1+0.001xFc^2^λ^3^/sin(2θ)]^-1/4^
Primary atom site location: structure-invariant direct methods	Extinction coefficient: 0.0012 (2)

### Special details

**Table:**

Geometry. All esds (except the esd in the dihedral angle between two l.s. planes) are estimated using the full covariance matrix. The cell esds are taken into account individually in the estimation of esds in distances, angles and torsion angles; correlations between esds in cell parameters are only used when they are defined by crystal symmetry. An approximate (isotropic) treatment of cell esds is used for estimating esds involving l.s. planes.
Refinement. Refinement of F^2^ against ALL reflections. The weighted R-factor wR and goodness of fit S are based on F^2^, conventional R-factors R are based on F, with F set to zero for negative F^2^. The threshold expression of F^2^ > 2sigma(F^2^) is used only for calculating R-factors(gt) etc. and is not relevant to the choice of reflections for refinement. R-factors based on F^2^ are statistically about twice as large as those based on F, and R- factors based on ALL data will be even larger.

### Fractional atomic coordinates and isotropic or equivalent isotropic displacement parameters (Å^2^)

**Table:**

	*x*	*y*	*z*	*U*_iso_*/*U*_eq_	
N1	0.04907 (16)	0.77988 (6)	0.34667 (4)	0.0461 (3)	
N2	0.03430 (17)	0.83315 (7)	0.30758 (4)	0.0501 (3)	
N3	0.1596 (2)	0.59304 (10)	0.21580 (6)	0.0737 (4)	
O1	0.05198 (19)	0.78631 (8)	0.17444 (4)	0.0747 (4)	
C1	0.06117 (18)	0.79131 (8)	0.26452 (5)	0.0480 (3)	
C2	0.09573 (19)	0.71052 (8)	0.27627 (5)	0.0463 (3)	
C3	0.08526 (18)	0.70532 (8)	0.32961 (5)	0.0441 (3)	
C4	0.1299 (2)	0.64619 (10)	0.24210 (5)	0.0536 (4)	
C5	0.0947 (2)	0.63535 (8)	0.36354 (5)	0.0458 (3)	
C6	0.2582 (2)	0.58729 (10)	0.36412 (6)	0.0628 (4)	
H6A	0.3661	0.5995	0.3436	0.075*	
C7	0.2603 (3)	0.52048 (10)	0.39553 (8)	0.0783 (5)	
H7A	0.3702	0.4880	0.3961	0.094*	
C8	0.1016 (4)	0.50213 (10)	0.42561 (7)	0.0785 (6)	
H8A	0.1043	0.4575	0.4467	0.094*	
C9	−0.0597 (3)	0.54911 (10)	0.42477 (7)	0.0741 (5)	
H9A	−0.1675	0.5363	0.4451	0.089*	
C10	−0.0647 (2)	0.61553 (9)	0.39400 (6)	0.0580 (4)	
H10A	−0.1758	0.6473	0.3937	0.070*	
C11	0.0388 (2)	0.80605 (8)	0.39950 (5)	0.0466 (3)	
C12	0.1957 (2)	0.79135 (9)	0.43180 (6)	0.0566 (4)	
H12A	0.3064	0.7656	0.4194	0.068*	
C13	0.1858 (3)	0.81549 (10)	0.48280 (6)	0.0650 (4)	
H13A	0.2901	0.8055	0.5050	0.078*	
C14	0.0225 (3)	0.85426 (9)	0.50097 (6)	0.0658 (4)	
H14A	0.0164	0.8703	0.5354	0.079*	
C15	−0.1319 (3)	0.86926 (10)	0.46807 (6)	0.0662 (4)	
H15A	−0.2414	0.8961	0.4803	0.079*	
C16	−0.1254 (2)	0.84480 (9)	0.41697 (6)	0.0576 (4)	
H16A	−0.2302	0.8544	0.3948	0.069*	
C17	0.0492 (2)	0.82851 (10)	0.21251 (5)	0.0568 (4)	
C18	0.0336 (3)	0.91591 (11)	0.20952 (7)	0.0755 (5)	
H18A	−0.0431	0.9303	0.1798	0.113*	
H18C	0.1621	0.9384	0.2065	0.113*	
H18D	−0.0284	0.9356	0.2403	0.113*	

### Atomic displacement parameters (Å^2^)

**Table:**

	*U*^11^	*U*^22^	*U*^33^	*U*^12^	*U*^13^	*U*^23^
N1	0.0550 (6)	0.0450 (6)	0.0383 (5)	−0.0011 (5)	−0.0011 (4)	0.0056 (4)
N2	0.0541 (6)	0.0525 (6)	0.0438 (6)	−0.0019 (5)	−0.0021 (5)	0.0112 (5)
N3	0.0732 (9)	0.0852 (10)	0.0626 (8)	0.0025 (8)	0.0026 (7)	−0.0182 (8)
O1	0.0859 (8)	0.0965 (9)	0.0417 (6)	−0.0136 (7)	−0.0014 (5)	0.0109 (6)
C1	0.0429 (6)	0.0603 (8)	0.0407 (7)	−0.0073 (6)	−0.0012 (5)	0.0096 (6)
C2	0.0429 (6)	0.0566 (8)	0.0395 (6)	−0.0071 (6)	0.0003 (5)	0.0014 (6)
C3	0.0446 (6)	0.0474 (7)	0.0404 (6)	−0.0036 (5)	0.0002 (5)	0.0014 (5)
C4	0.0479 (7)	0.0701 (9)	0.0429 (7)	−0.0050 (7)	0.0009 (6)	−0.0026 (7)
C5	0.0579 (7)	0.0430 (7)	0.0363 (6)	−0.0014 (6)	−0.0021 (5)	−0.0020 (5)
C6	0.0642 (9)	0.0621 (9)	0.0622 (9)	0.0079 (7)	−0.0024 (7)	−0.0025 (7)
C7	0.0960 (13)	0.0558 (9)	0.0832 (12)	0.0250 (9)	−0.0237 (11)	−0.0051 (9)
C8	0.1315 (17)	0.0486 (9)	0.0554 (9)	0.0027 (10)	−0.0064 (10)	0.0067 (7)
C9	0.1125 (14)	0.0530 (9)	0.0568 (9)	−0.0034 (9)	0.0177 (9)	0.0085 (7)
C10	0.0738 (9)	0.0493 (8)	0.0508 (8)	0.0007 (7)	0.0113 (7)	0.0040 (6)
C11	0.0603 (7)	0.0395 (6)	0.0400 (6)	−0.0025 (6)	−0.0011 (6)	0.0034 (5)
C12	0.0651 (9)	0.0520 (8)	0.0526 (8)	0.0055 (7)	−0.0077 (6)	−0.0043 (6)
C13	0.0863 (11)	0.0575 (9)	0.0511 (8)	0.0016 (8)	−0.0186 (8)	−0.0037 (7)
C14	0.0962 (12)	0.0568 (9)	0.0445 (8)	−0.0048 (8)	−0.0002 (8)	−0.0051 (7)
C15	0.0801 (10)	0.0616 (9)	0.0571 (9)	0.0084 (8)	0.0100 (8)	−0.0040 (7)
C16	0.0650 (9)	0.0580 (8)	0.0499 (8)	0.0069 (7)	−0.0005 (6)	0.0038 (7)
C17	0.0460 (7)	0.0792 (10)	0.0451 (8)	−0.0084 (7)	−0.0012 (6)	0.0152 (7)
C18	0.0816 (11)	0.0821 (12)	0.0627 (10)	0.0030 (9)	0.0005 (8)	0.0291 (9)

### Geometric parameters (Å, º)

**Table:**

N1—N2	1.3555 (15)	C9—C10	1.375 (2)
N1—C3	1.3572 (17)	C9—H9A	0.9300
N1—C11	1.4346 (17)	C10—H10A	0.9300
N2—C1	1.3294 (18)	C11—C16	1.375 (2)
N3—C4	1.144 (2)	C11—C12	1.380 (2)
O1—C17	1.214 (2)	C12—C13	1.379 (2)
C1—C2	1.418 (2)	C12—H12A	0.9300
C1—C17	1.4840 (18)	C13—C14	1.376 (3)
C2—C3	1.3807 (18)	C13—H13A	0.9300
C2—C4	1.419 (2)	C14—C15	1.378 (2)
C3—C5	1.4724 (18)	C14—H14A	0.9300
C5—C6	1.381 (2)	C15—C16	1.382 (2)
C5—C10	1.384 (2)	C15—H15A	0.9300
C6—C7	1.390 (2)	C16—H16A	0.9300
C6—H6A	0.9300	C17—C18	1.483 (2)
C7—C8	1.369 (3)	C18—H18A	0.9600
C7—H7A	0.9300	C18—H18C	0.9600
C8—C9	1.359 (3)	C18—H18D	0.9600
C8—H8A	0.9300		
			
N2—N1—C3	112.88 (11)	C9—C10—H10A	119.8
N2—N1—C11	119.90 (10)	C5—C10—H10A	119.8
C3—N1—C11	127.08 (11)	C16—C11—C12	121.44 (13)
C1—N2—N1	104.96 (11)	C16—C11—N1	119.87 (12)
N2—C1—C2	110.88 (11)	C12—C11—N1	118.69 (12)
N2—C1—C17	121.50 (13)	C13—C12—C11	118.99 (15)
C2—C1—C17	127.60 (13)	C13—C12—H12A	120.5
C3—C2—C1	105.40 (12)	C11—C12—H12A	120.5
C3—C2—C4	125.39 (13)	C14—C13—C12	120.36 (15)
C1—C2—C4	129.20 (13)	C14—C13—H13A	119.8
N1—C3—C2	105.88 (11)	C12—C13—H13A	119.8
N1—C3—C5	124.11 (11)	C13—C14—C15	119.91 (14)
C2—C3—C5	129.87 (12)	C13—C14—H14A	120.0
N3—C4—C2	177.90 (16)	C15—C14—H14A	120.0
C6—C5—C10	119.21 (14)	C14—C15—C16	120.54 (15)
C6—C5—C3	120.93 (13)	C14—C15—H15A	119.7
C10—C5—C3	119.83 (12)	C16—C15—H15A	119.7
C5—C6—C7	119.48 (16)	C11—C16—C15	118.75 (15)
C5—C6—H6A	120.3	C11—C16—H16A	120.6
C7—C6—H6A	120.3	C15—C16—H16A	120.6
C8—C7—C6	120.43 (17)	O1—C17—C18	122.97 (14)
C8—C7—H7A	119.8	O1—C17—C1	118.81 (15)
C6—C7—H7A	119.8	C18—C17—C1	118.22 (14)
C9—C8—C7	120.07 (16)	C17—C18—H18A	109.5
C9—C8—H8A	120.0	C17—C18—H18C	109.5
C7—C8—H8A	120.0	H18A—C18—H18C	109.5
C8—C9—C10	120.41 (18)	C17—C18—H18D	109.5
C8—C9—H9A	119.8	H18A—C18—H18D	109.5
C10—C9—H9A	119.8	H18C—C18—H18D	109.5
C9—C10—C5	120.39 (16)		
			
C3—N1—N2—C1	0.20 (14)	C5—C6—C7—C8	0.2 (3)
C11—N1—N2—C1	176.31 (11)	C6—C7—C8—C9	0.3 (3)
N1—N2—C1—C2	−0.67 (14)	C7—C8—C9—C10	−0.4 (3)
N1—N2—C1—C17	177.93 (11)	C8—C9—C10—C5	0.0 (3)
N2—C1—C2—C3	0.89 (15)	C6—C5—C10—C9	0.5 (2)
C17—C1—C2—C3	−177.61 (12)	C3—C5—C10—C9	178.21 (14)
N2—C1—C2—C4	179.54 (13)	N2—N1—C11—C16	59.51 (17)
C17—C1—C2—C4	1.0 (2)	C3—N1—C11—C16	−124.99 (15)
N2—N1—C3—C2	0.34 (14)	N2—N1—C11—C12	−120.59 (14)
C11—N1—C3—C2	−175.42 (12)	C3—N1—C11—C12	54.91 (19)
N2—N1—C3—C5	−175.66 (11)	C16—C11—C12—C13	0.7 (2)
C11—N1—C3—C5	8.6 (2)	N1—C11—C12—C13	−179.21 (13)
C1—C2—C3—N1	−0.71 (14)	C11—C12—C13—C14	−0.6 (2)
C4—C2—C3—N1	−179.43 (12)	C12—C13—C14—C15	−0.1 (3)
C1—C2—C3—C5	174.97 (13)	C13—C14—C15—C16	0.8 (3)
C4—C2—C3—C5	−3.7 (2)	C12—C11—C16—C15	0.0 (2)
N1—C3—C5—C6	−124.42 (15)	N1—C11—C16—C15	179.86 (14)
C2—C3—C5—C6	60.6 (2)	C14—C15—C16—C11	−0.7 (3)
N1—C3—C5—C10	57.90 (18)	N2—C1—C17—O1	−171.47 (13)
C2—C3—C5—C10	−117.09 (16)	C2—C1—C17—O1	6.9 (2)
C10—C5—C6—C7	−0.5 (2)	N2—C1—C17—C18	8.3 (2)
C3—C5—C6—C7	−178.24 (14)	C2—C1—C17—C18	−173.32 (14)

### Hydrogen-bond geometry (Å, º)

**Table:**

*D*—H···*A*	*D*—H	H···*A*	*D*···*A*	*D*—H···*A*
C6—H6*A*···N3^i^	0.93	2.53	3.432 (2)	165
C16—H16*A*···O1^ii^	0.93	2.59	3.3758 (19)	142

Symmetry codes: (i) *x*+1/2, *y*, −*z*+1/2; (ii) *x*−1/2, *y*, −*z*+1/2.
